# Improvement of Shen’ge formula on heart function in diastolic heart failure: A protocol for randomized, double-blind, placebo-controlled clinical study

**DOI:** 10.1097/MD.0000000000025383

**Published:** 2021-04-02

**Authors:** Boyong Qiu, Ping Zhao, Lin Shen, Siyu Qiao, Guanghao Li, Bing Deng, Duan Zhou, Yihong Wei

**Affiliations:** Department of cardiology, Longhua Hospital affiliated to Shanghai University of Traditional Chinese Medicine. Shanghai, PR China.

**Keywords:** diastolic heart failure, randomized controlled trial, Shen’ge formula, traditional Chinese medicine

## Abstract

**Introduction:**

Diastolic heart failure (DHF) is an important pathological type of heart failure, that involves multiple organ dysfunction and multiple complications. The prevalence of DHF is high, and effective treatments are lacking. Chinese herbs are an alternative therapy for DHF. Shen’ge formula (SGF) is a classical formula from which patients can benefit, but convincing evidence of its efficacy is lacking. Therefore, we designed this randomized controlled trial protocol.

**Methods/design:**

This randomized, double-blind, placebo-controlled clinical trial will evaluate the efficacy and safety of SGF in the treatment of DHF. A total of 130 patients with DHF will be enrolled in the trial and treated with SGF granules or placebo for 12 weeks and followed up for 12 weeks. The primary outcome measurement will be to changes in plasma N-terminal brain natriuretic peptide precursor before versus after treatment, while the second primary outcome measurement will be changes in heart function before versus after treatment and the 12-week follow-up period. It will also include echocardiography, a cardiopulmonary exercise test, cardiac function grading, traditional Chinese medicine syndrome score, and the Minnesota Heart Failure Quality of Life Scale. Adverse events will be evaluated throughout the trial.

**Discussion:**

The results of this trial will demonstrate whether SGF could alleviate symptoms, improve cardiac function, reduce readmission rates, and improve quality of life of patients with DHF.

**Trial registration:**

Chinese Clinical Trial Register, ChiCTR2000036533, registered on August 24, 2020.

## 1 Introduction

Diastolic heart failure (DHF) is also known as heart failure (HF) with preserved ejection fraction (HFpEF). The prevalence of DHF has been increasing at a rate of 1% per year, increasing from 38% to 54% over the past 20 years.^[1]^ The data collected from the American College of Cardiology's National Cardiovascular Data Registry Practice Innovation and Clinical Excellence showed that 56.5% (n = 622 866) of 1 103 386 patients had HFpEF.^[2]^ A study also showed that more than 90% of patients had HF with diastolic dysfunction independent of ejection fraction.^[3]^ However, as a current major public health problem, there is no effective treatment for DHF,^[4]^ and the interventions mainly include smoking cessation, moderate exercise, weight control, and the treatment of basic diseases.^[5]^ Moreover, DHF will be a major cause of HF in the next 10 years and involves multiple organ dysfunction with multiple complications.^[6]^

Traditional Chinese medicine (TCM), a verified complementary or alternative treatment for patients with HF,^[7–11]^ has been used to treat various heart diseases in China for thousands of years. In our study, Shen’ge formula (SGF), a representative TCM formula, consisting of ginseng and gecko (Table 1) in a 3:1 proportion by weight will be used as a therapeutic drug to treat patients with DHF. A previous study showed that SGF could improve cardiac function and myocardial contractility in HF patients.^[12]^ Gu Ben Pei Yuan San, another TCM formula containing ginseng, gecko, and other herbs, was suggested to improve cardiac function and repair the myocardium in adult mice after apical resection or myocardial infarction.^[13]^ To promote the use of SGF for the treatment of DHF worldwide, randomized, double-blind, placebo-controlled clinical trials are still needed to prove its effectiveness. All of the information for this study was obtained from the Chinese Clinical Trial Registry (registration number: ChiCTR2000036533; http://www.chictr.org.cn).

**Table 1:** Components of SGF.

Latin scientific name	English name	Chinese name
*Panax ginseng C.A.Mey.*	Ginseng	Renshen
*Gekko gecko Linnaeus*	Gecko	Gejie

SGF = Shen’ge formula.

This randomized double-blind placebo-controlled clinical trial of DHF will include 12 weeks of clinical intervention and 12 weeks of follow-up. Changes in plasma N-terminal brain natriuretic peptide precursor (NT-proBNP) before versus after treatment will be the primary outcome measure. Changes in cardiac ultrasound, cardiopulmonary exercise test, cardiac function grade, TCM syndrome score and Minnesota Heart Failure Quality of Life Scale will be the secondary outcome measure. We will also assess its safety aspects by thoroughly documenting any adverse events (AEs) that occur.

## 2 Methods

### 2.1 Study design

**Figure 1:**
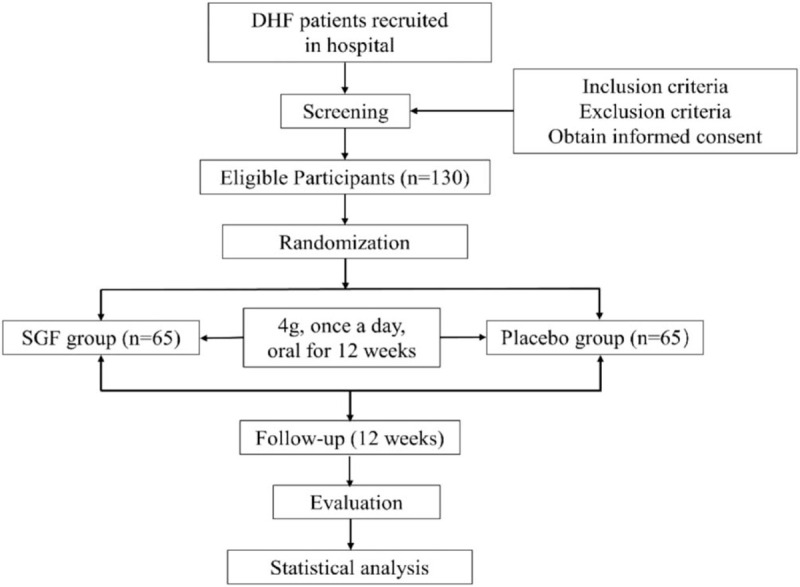
Study flowchart. The entire trial will last 24 weeks including 12 weeks for oral drugs and another 12 weeks for follow-up. DHF = diastolic heart failure, SGF = Shen’ge formula.

This randomized double-blind placebo-controlled clinical trial will investigate the efficacy and safety of SGF in DHF patients. We will recruit 130 patients with DHF from LongHua Hospital affiliated with the Shanghai University of TCM. A total of 130 participants will be equally randomized into the SGF or placebo group. Patients in the SGF group will take 4 g SGF per day for 12 weeks, while patients in the placebo group will take SGF placebo for the same duration. The trial process is summarized in Figure 1.

### 2.2 Ethical issues

The clinical trial will follow the Declaration of Helsinki and the Ethical Guidelines for Clinical Research. The trial protocol was approved by the Institutional Review Board of Longhua Hospital affiliated with Shanghai University of Traditional Chinese Medicine (approval number: 2020LHSB053). Additionally, the protocol has been registered in the Chinese Clinical Trial Registry (ChiCTR2000036533). Personal information about potential and registered participants will be collected for use only in this trial, and we will not share or retain such personal information when it is not necessary.

### 2.3 Study participants

Participants will be recruited from LongHua Hospital affiliated with Shanghai University of Traditional Chinese Medicine. Recruitment posters and social application advertisements will be distributed. The objective, approaches, and potential side effects and advantages of this study will be fully explained in writing to the patient and his or her family or designated representative. Before entering the trial, patients and their families must provide written informed consent, and patients and their families have the right to withdraw from the study at any time. Informed consent will be retained as clinical trial documents for future reference. The planned enrollment period is 24 months.

### 2.4 Main inclusion criteria

(1) Patients who meet the diagnostic criteria of Western medicine and TCM;
(2) Patients with New York Heart Association (NYHA) grade II–IV cardiac function;
(3) Patients aged 18 to 80 years;
(4) Patients who are not taking forbidden medicine and are able to complete the trial; and
(5) Patients who participate in this clinical trial voluntarily, understand its objectives, and provide written informed consent.

### 2.5 Main exclusion criteria

1. Shock, acute myocardial infarction, pulmonary embolism, infectious endocarditis, severe hepatic, or renal insufficiency;
2. Currently pregnant or lactating or current mental disorder;
3. Seriously ill patients with a life expectancy of less than 6 months; or
4. Patients who are taking other TCM and unwilling to stop.

### 2.6 Randomization and allocation

Using the random number method in Excel software, the random arrangement of treatment (therapeutic versus control drug) received by 130 subjects will be generated by the given seed number of 2020, and the corresponding treatment allocation for the serial number of 1 to 130 will be listed. According to the order in which the subjects are enrolled, the researchers will fill in a random number on the medical record sheet and distribute the experimental drugs matching the random number.

### 2.7 Blinding

TCM granules (SGF) and placebo will be packaged identically. The label on each package includes the drug number, quantity, method of administration, storage conditions, the label “for clinical research only,” and the drug supply unit. The 2 drugs will be randomly cataloged, and the doctors in charge of the general manager plan will issue medical advice for drug distribution, the nurses will issue the drugs, and the doctors in charge of the patients will register the drug distribution. The doctors and nurses in charge of the patients will be blinded to the patients’ drug allocations.

### 2.8 Interventions

#### 2.8.1 TCM intervention

In the SGF group, patients will be instructed to dissolve SGF (4 g) in 50 mL of hot water and take the solution orally once a day for 12 weeks, while patients in the placebo group will take SGF placebo in the same way. The SGF and SGF placebo will be manufactured, packaged, and labeled by the Department of Pharmacy, Longhua Hospital Affiliated to Shanghai University of Chinese Medicine. SGF consists of ginseng (Renshen, 3 g) and gecko (Gejie 1 g). All herbs will be crushed into a powder using a pulverizer and collected after being subjected to No. 10 sifter-mediated selection. Finally, the SGF will be packed in individual bags (4 g each). For the control group, SGF placebo will also be prepared by the above department to achieve a similar color, smell, taste, and texture as the SGF. The research drugs will be stored in cool and dark places, placed in dry places away from light, managed and distributed by special personnel, and tracked carefully.

#### 2.8.2 Conventional Western medicine intervention

All enrolled patients will be administered basic drug therapy according to the Chinese Guidelines for the Diagnosis and Treatment of Heart Failure, Chinese Society of Cardiology (2018). Angiotensin-converting enzyme inhibitors or angiotensin receptor blockers, β-receptor blockers, diuretics, digitalis drugs, and nitrate drugs will be selected for specific situations.

#### 2.8.3 Forbidden treatments and drugs

(1) Any other TCM methods are not allowed (herbs except for SGF or SGF placebo, acupuncture, cupping, etc).
(2) Medications outside of the guidelines are not allowed.
(3) The dosage, duration, and name of any intervention must be recorded thoroughly in the case report form.

### 2.9 Outcomes

#### 2.9.1 Primary outcomes

NT-proBNP levels will be determined using an enzyme-linked immunosorbent assay kit before and after drug administration (week 0 and week 12).

#### 2.9.2 Secondary outcomes

##### 2.9.2.1 Echocardiography

Echocardiography includes left ventricular mass index, E/E′ ratio, pulmonary systolic pressure, and right ventricular end-diastolic volume.

##### 2.9.2.2 Cardiopulmonary exercise test

Peak oxygen uptake, anaerobic threshold, and other indicators will be detected at weeks 0 and 12.

##### 2.9.2.3 NYHA cardiac function classification

NYHA cardiac function classification: Cardiac function I, patients with heart disease but unrestricted activity in whom normal physical activity does not cause fatigue, palpitations, shortness of breath, or angina. Cardiac function II, heart function and physical activity are limited by mild heart attack, no discomfort when resting but normal activity can lead to fatigue, heart palpitations, shortness of breath, or angina. Cardiac function III, heart disease, limited physical activity, less than the usual general activity causes symptoms. Cardiac function IV, heart patients cannot perform any physical activity, and in the resting state can experience symptoms of HF or angina pectoris plus aggravating discomfort after physical activity.

Significant effect: Cardiac function was restored to level I or improved by 2 levels.

Effective: Cardiac function improves by 1 but not 2 levels.

No effect: No change in cardiac function; deterioration; cardiac function decreased by 1 or more levels.

### 2.10 TCM syndrome score

This score form is constitutive of 2 parts: symptoms and signs. There are a total of nine appraisable items including palpitation, shortness of breath, lassitude, asthma, felling cold, chest distress, edema, oliguria, and abdominal distention. Each item consists of 4 levels. The minimum score is 0, and the maximum score is 54. Higher scores indicate more severe conditions. The scores and details of TCM syndromes are presented in Table 2. The scores are determined using the semi-quantitative integral method (0–6 points) according to the severity of clinical symptoms.

**Table 2:** Traditional Chinese medicine syndrome score.

Syndromes	None	Mild	Moderate	Severe
Palpitation	None	Slight palpitations during activities; able to work	Obvious palpitations during activities; relief after rest	Unable to join routine activities
Shortness of breath	No syndrome	Short of breath after slight activity	Short of breath after moderate activity	Short of breath during rest
Fatigued	None	Slight fatigue; able to work	Heavy and barely able to work or complete routine activities	Too heavy to work or complete routine activities
Asthma	None	Slight wheezing; able to work	Frequent wheezing; able to sleep	Unable to lie down and sleep
Felling cold	None	Fear cold	Need to add clothes	Still feel cold after adding clothes
Chest distress	None	Slight	Often	Always
Edema	None	Slight edema morning and night	Pitting edema (+to++)	Pitting edema (>++)
Oliguria	None	Little urine (>1000 mL within 24 h)	Little urine (<400 mL within 24 h)	Little urine (<100 mL within 24 h)
Abdominal distention	None	Slight	Obvious	Like bulge and cannot press

Scores: Mild = 2, Moderate = 4, None = 0, Severe = 6.

Significant effect: Clinical primary and secondary symptoms disappeared partially or completely, syndrome score decreased by > 70%. Effectiveness: Clinical symptoms improved significantly, syndrome scores decreased by 30% to 70%. Null: Treatment syndrome score decreased by < 30%. Aggravation: Post-treatment points exceed pre-treatment points.

### 2.11 Minnesota Heart Failure Quality of Life Scale

The Minnesota Heart Failure Quality of Life Scale is widely used to measure the impact of HF on life.^[14]^ This scale involves the impact of HF on patient lifestyle within the last 1 month (Table 3).

**Table 3:** Minnesota heart failure quality of life scale.

Did your heart failure prevent you from living as you wanted during the last month by	No	Very Little				Very Much
1. Causing swelling in your ankles or legs, etc?	0	1	2	3	4	5
2. Making you sit or lie down to rest during the day?	0	1	2	3	4	5
3. Making your walking about or climbing stairs difficult?	0	1	2	3	4	5
4. Making your working around the house or yard difficult?	0	1	2	3	4	5
5. Making your going places away from home difficult?	0	1	2	3	4	5
6. Making your sleeping well at night difficult?	0	1	2	3	4	5
7. Making your relating to or doing things with your friends or family difficult?	0	1	2	3	4	5
8. Making your working to earn a living difficult?	0	1	2	3	4	5
9. Making your recreational pastimes, sports or hobbies difficult?	0	1	2	3	4	5
10. Making your sexual activities difficult?	0	1	2	3	4	5
11. Making you eat less of the foods you like?	0	1	2	3	4	5
12. Making you short of breath?	0	1	2	3	4	5
13. Making you tired, fatigued, or low on energy?	0	1	2	3	4	5
14. Making you stay in a hospital?	0	1	2	3	4	5
15. Costing you money for medical acre?	0	1	2	3	4	5
16. Giving you side effects from treatments?	0	1	2	3	4	5
17. Making you feel you are a burden to your family or friends?	0	1	2	3	4	5
18. Making you feel a loss of self-control in your life?	0	1	2	3	4	5
19. Making you worry?	0	1	2	3	4	5
20. Making it difficult for you to concentrate or remember things?	0	1	2	3	4	5
21. Making you feel depressed?	0	1	2	3	4	5

### 2.12 Safety assessments

AE time, severity, duration, action taken, and outcomes will be recorded truthfully on the designated case report forms (CRFs) during the study. Mild, moderate, and severe labels will be used to describe the intensity of each AE and assess possible associations between each AE and the study or control drug. If a severe AE occurs during the trial, the patients will be suspended from the trial and treated as a lost case. For the safety assessment, the patients’ blood, urine, and kidney and liver function will be tested before and after treatment. To protect privacy, all participants will be visited in a closed room and given the option to withdraw at any time.

### 2.13 Sample size

One study^[15]^ showed that HF patients whose NT-proBNP levels decreased by more than 30% had better clinical prognosis. In this study, it was estimated that the decrease in plasma NT-proBNP in the treatment group after treatment would reach 42%. Formula n^[16]^$n={\left[\frac{\left(u_{\alpha }+u_{\beta }\right)}{\delta /\sigma }\right]}^{2}+\frac{1}{2}u_{\alpha }^{2}$ was calculated according to the sample size, where standard deviation (instead of σ) is set to 30%, α = 0.05, uα = 1.645, β = 0.1, uβ = 1.282, and δ = 42–30 = 12.

The sample size was calculated as 54 cases, considering a withdrawal rate of no more than 20%, the sample size of this study was determined to be 130 people with a ratio of the experimental and control groups of 1:1 for 65 people in each group.

### 2.14 Data collection and monitoring

In this 24-week trial, all participants will be treated with SGF or SGF placebo for 12 weeks and followed up for another 12 weeks. All data will be entered into the designated database that will be exclusively managed by the research team members. Electronic data files, including databases, analysis programs, analysis results, and explanatory files, will be classified and stored in multiple backups on different recording media and properly stored to prevent damage. All of the original files shall be kept in accordance with the unified requirements of scientific research management, and the management data shall be transmitted in time.

### 2.15 Quality control

Longhua Hospital affiliated with Shanghai University of Traditional Chinese Medicine (http://www.longhua.net/ywsy/gzlc/287.jhtml) is responsible for quality control and training for all investigators. All team members of the trial will receive systematic training to completely apprehend the study procedures. The person in charge will regularly inspect the subjects, identify the problem, corrects it in time, and cancel a researcher's qualification when the problem is serious. The monitor will regularly monitor the study and discuss with the investigator its progress, the CRF and the original records, and the accuracy of the data records.

### 2.16 Statistical analysis

The statistical analysis will be performed using SPSS 20.0. If the measurement data are normally distributed with homogeneous variance, the mean ± standard (± s) will be used for the statistical analysis and the t-test will be used for the intergroup comparisons. If the data do not follow a normal distribution, the rank sum test will be used to make statistical inferences. Enumeration data will be statistically described as frequency, constituent ratio, and rate. Comparison of indicators with dichotomous or multi-classification disorders will be performed using the *χ*^2^ test. The rank sum test will be used to compare groups of ordered classification analysis indicators. Statistical significance will be set at *P* < .05.

## 3 Discussion

HF, the final stage of many heart diseases, is a worldwide problem. The report mentioned that mortality, hospitalization, and rehospitalization rates remained high in HF patients.^[17]^ The 2016 ESC Guidelines^[18]^ highlighted the ejection fraction preservation in the diagnosis of HF, suggesting that we must pay more attention to the status of patients with DHF. There is currently no clear and effective drug for the treatment of DHF, and high-quality clinical trials on this disease treated by TCM are lacking. SGF is a TCM that has been used for hundreds of years owing to its unique theoretical connotation and practical effect, but its clinical efficacy and safety have not been systematically evaluated in randomized controlled trials.

A query of PubMed, Web of Science, Embase, the Cochrane Library, Wan Fang Database, and Clinical Trials for publications through February 2021 revealed no data about the efficacy or safety of SGF for treating DHF patients. Therefore, this study will be the first randomized double-blind placebo-controlled study to evaluate the efficacy and safety of the SGF in the treatment of patients with DHF.

The results of this study will not only prove the exact efficacy of SGF on DHF, also it will document changes in SGF on quality of life through follow-up. This clinical trial will provide powerful evidence to provide a safe and effective reference for future research.

## Acknowledgments

We deeply thank all patients who will participate in this trial and all the staff for their kind support.

## Author contributions

**Conceptualization:** Boyong Qiu, Ping Zhao.

**Investigation:** Lin Shen, Guanghao Li.

**Methodology:** Lin Shen.

**Resources:** Yihong Wei.

**Software:** Boyong Qiu, Duan Zhou.

**Supervision:** Bing Deng, Duan Zhou.

**Visualization:** Bing Deng.

**Writing – original draft:** Boyong Qiu, Siyu Qiao.

**Writing – review & editing:** Yihong Wei.
